# iTRAQ-Based Protein Profiling and Biochemical Analysis of Two Contrasting Rice Genotypes Revealed Their Differential Responses to Salt Stress

**DOI:** 10.3390/ijms20030547

**Published:** 2019-01-28

**Authors:** Sajid Hussain, Chunquan Zhu, Zhigang Bai, Jie Huang, Lianfeng Zhu, Xiaochuang Cao, Satyabrata Nanda, Saddam Hussain, Aamir Riaz, Qingduo Liang, Liping Wang, Yefeng Li, Qianyu Jin, Junhua Zhang

**Affiliations:** 1 State Key Laboratory of Rice Biology, China National Rice Research Institute, Hangzhou 310006, Zhejiang, China; sajid_2077uaf@yahoo.com (S.H.); zhuchunquan@caas.cn (C.Z.); baizg1989@163.com (Z.B.); huangjie67179484@163.com (J.H.); zlfnj@163.com (L.Z.); caoxiaochuang@126.com (X.C.); sbn.satyananda@gmail.com (S.N.); aamirriaz33@gmail.com (A.R.); 15550883578@163.com (Q.L.); 664948431@163.com (L.W.); m13067998118@163.com (Y.L.); 2 Department of Agronomy, University of Agriculture Faisalabad, Punjab 38000, Pakistan; sadamhussainuaf@gmail.com

**Keywords:** Salt stress, *Oryza sativa*, proteomics, iTRAQ quantification, cell membrane injury, root activity

## Abstract

Salt stress is one of the key abiotic stresses causing huge productivity losses in rice. In addition, the differential sensitivity to salinity of different rice genotypes during different growth stages is a major issue in mitigating salt stress in rice. Further, information on quantitative proteomics in rice addressing such an issue is scarce. In the present study, an isobaric tags for relative and absolute quantitation (iTRAQ)-based comparative protein quantification was carried out to investigate the salinity-responsive proteins and related biochemical features of two contrasting rice genotypes—Nipponbare (NPBA, *japonica*) and Liangyoupeijiu (LYP9, *indica*), at the maximum tillering stage. The rice genotypes were exposed to four levels of salinity: 0 (control; CK), 1.5 (low salt stress; LS), 4.5 (moderate salt stress; MS), and 7.5 g of NaCl/kg dry soil (high salt stress, HS). The iTRAQ protein profiling under different salinity conditions identified a total of 5340 proteins with 1% FDR in both rice genotypes. In LYP9, comparisons of LS, MS, and HS compared with CK revealed the up-regulation of 28, 368, and 491 proteins, respectively. On the other hand, in NPBA, 239 and 337 proteins were differentially upregulated in LS and MS compared with CK, respectively. Functional characterization by KEGG and COG, along with the GO enrichment results, suggests that the differentially expressed proteins are mainly involved in regulation of salt stress responses, oxidation-reduction responses, photosynthesis, and carbohydrate metabolism. Biochemical analysis of the rice genotypes revealed that the Na^+^ and Cl^−^ uptake from soil to the leaves via the roots was increased with increasing salt stress levels in both rice genotypes. Further, increasing the salinity levels resulted in increased cell membrane injury in both rice cultivars, however more severely in NPBA. Moreover, the rice root activity was found to be higher in LYP9 roots compared with NPBA under salt stress conditions, suggesting the positive role of rice root activity in mitigating salinity. Overall, the results from the study add further insights into the differential proteome dynamics in two contrasting rice genotypes with respect to salt tolerance, and imply the candidature of LYP9 to be a greater salt tolerant genotype over NPBA.

## 1. Introduction

To satisfy the food demands of a population of more than nine billion people by 2050, the world’s food productivity needs to be increased by 50% above current production [1,2]. The current growth trends of the major food crops, including wheat, rice, maize, and soybean, suggest that crop production will not be sufficient to meet these ever-rising food demands [3]. Further, the occurrence of abiotic stresses owing to climate change is one of the major reasons for the productivity gap [4]. Soil salinity is considered to be a major problem in the productivity of rice (*Oryza sativa* L.) worldwide [4]. Rice is highly sensitive to salt stress; however, the range of sensitivity varies with rice ecotypes, genotypes, and growth stages [5,6]. Salt tolerance in rice is correlated with variations in the translocation of sodium (Na^+^) and chloride (Cl^−^) ions in the aboveground plant organs, including the shoot and panicles [7,8,9,10,11,12]. Salinity affects rice physiology and growth by causing osmotic stress, nutrient imbalance, ionic toxicity, oxidative damage, alteration of metabolic processes, reduced cell division, genotoxicity, decline of growth and yield, and even the death of the plant [8,9,13,14,15,16,17]. In rice, salinity tolerance is usually achieved as a result of a cocktail of physiological and genetic reprogramming, including selective ion uptake and exclusion, preferential compartmentation of Na^+^, alternation in stomatal closure, reactive oxygen species (ROS) signaling, and expression of salt-stress responsive genes and transcription factors [18,19,20,21,22].

Alterations in physiological and biochemical processes lead to changes in the protein pool in plants. In recent times, proteomic analysis has emerged as a significant molecular technique for the profiling and identification of proteins expressed in response to various abiotic stresses [23]. Isobaric tags for relative and absolute quantitation (iTRAQ)-based protein profiling and analysis has been performed in several crops, including rice [24], maize [25], wheat [26], tomatoes [27], and cotton [23], in response to abiotic stresses. Differential protein expressions in the areal tissues of rice subjected to salt stress have been reported by a few studies [28,29,30]. However, most of these studies have employed the 2D gel electrophoresis method to quantify the protein dynamics in rice. The 2D gel electrophoresis technique lacks efficiency in identifying the low abundant proteins, including extreme-acidic or basic proteins, proteins with molecular weights <15 kDa or >150 kDa, and hydrophobic proteins [23]. Furthermore, most of these works have been performed using the *japonica* rice genotype “Nipponbare” as the plant material. Therefore, in this study, we explored the proteomic dynamics of rice under salt stress in both *japonica* (Nipponbare, NPBA) and *indica* (Liangyoupeijiu, LYP9) rice genotypes by employing an iTRAQ-based proteomic study.

In the current study, the iTRAQ-based proteomic technique was used to identify the differentially expressed proteins in two rice genotypes of contrasting salt tolerance levels. The *indica* rice LYP9 has a higher salt tolerance level than the *japonica* rice NPBA [13]. Therefore, the proteomic analysis was performed with the aim of elucidating and comparing the effects of salt stress in these rice genotypes. Further, the physiological responses, such as cell membrane injury (CMI) and rice root activity of the NPBA and LYP9 genotypes, were assessed in response to various salt stress levels at the maximum tillering stage. Additionally, the Na^+^ and Cl^−^ uptake from soil to leaf via root under the subjected salt stress levels were determined in both rice genotypes. The results from this study will help us to achieve better insights into the salt stress resistance mechanisms in rice.

## 2. Results

### 2.1. Na^+^ and Cl^−^ in the Soil

The soil Na^+^ concentrations for LYP9 rice were recorded to be 0.17, 0.95, 1.7, and 2.0 mg·g^−1^ for the control (no salt stress, CK), low salt stress (LS), moderate salt stress (MS), and high salt stress (HS) treatments, respectively. In NPBA, the soil Na^+^ was recorded to be 0.18, 1.0, 1.6, and 2.15 mg·g^−1^ for the CK, LS, MS, and HS treatments, respectively. The Na^+^ concentration was found to be the highest in the HS treatment for NPBA rice, as most of the rice seedlings died under the HS condition before attaining the maximum tillering stage. Furthermore, the soil Na^+^ concentration was lower for the LYP9 rice than the NPBA rice (Table 1). On the other hand, the soil Cl^−^ concentrations were recorded to be 0.04, 0.59, 2.17, and 2.43 mg·g^−1^ for the CK, LS, MS, and HS treatments in LYP9 rice, respectively. In NPBA, the soil Cl^−^ was found to be 0.01, 0.66, 1.64, and 3.03 mg·g^−1^ for the CK, LS, MS, and HS treatments, respectively.

### 2.2. Na^+^ and Cl^−^ in the Rice Plants

The concentration of Na^+^ was found to increase in rice in proportion to rice growth. At the time of rice transplanting, the Na^+^ concentration in the LYP9 and NPBA roots was 0.44 and 0.37 mg·g^−1^, respectively. However, at the maximum tillering stage, Na^+^ concentrations in rice roots was increased in both rice genotypes, with the increase in subjected salt stress levels. In LYP9 rice, LS, MS, and HS levels of salt stress resulted in the increase of Na^+^ concentrations in rice roots amounting to 67.2%, 126.9%, and 138.8%, respectively, as compared with the CK treatment. Similarly, in NPBA rice, Na^+^ concentration in the roots was increased by 42.9% for LS and 128.6% for MS as compared with the CK treatment. However, the NPBA rice could not survive under HS salinity conditions. These results indicated that the uptake of Na^+^ is higher in rice in the maximum tillering stage as compared to the seedling stage (Table 1). Similar proportions were observed for Na^+^ concentration in rice leaves, where the Na^+^ concentrations were found to be increased by 163.2%, 305.3%, and 357.9% under LS, MS, and HS conditions, respectively, as compared with the CK condition in LYP9 rice, and by 86.7% and 480% under LS and MS conditions, respectively, as compared with the CK condition in NPBA rice (Table 1). The Na^+^ uptake from root to shoot was found to be higher in LYP9 than NPBA. These results suggest that LYP9 has an enhanced ability to uptake Na^+^ in the plant parts than compared to NPBA, which might aid in improved salt tolerance in LYP9 compared with NPBA. Likewise, at the maximum tillering stage, the Cl^−^ uptake by the rice roots and leaves was increased with the increase in the salt stress levels (Table 1). Moreover, these increases in the Cl^−^ ion uptakes were found to be higher in LYP9 leaves and roots than those of NPBA.

### 2.3. Cell Membrane Injury (CMI) in Rice Flag Leaves

**Figure 1:**
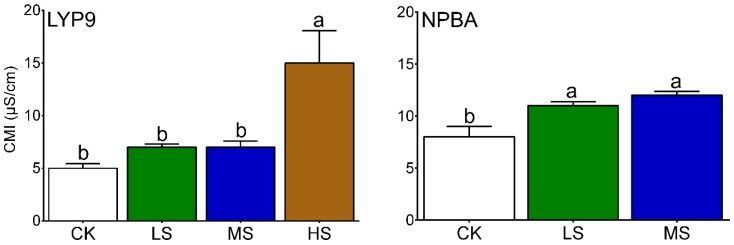
Evaluation of cell membrane injury under the subjected salt stress in LYP9 and NPBA. Bars denoted mean values ± SE (*n* = 3). Values followed by different letters denote significant difference (*p* ≤ 0.05) according to LSD test. The similar lettering within rice genotype shows the significant and different lettering mean non-significance within treatment levels.

**Figure 2:**
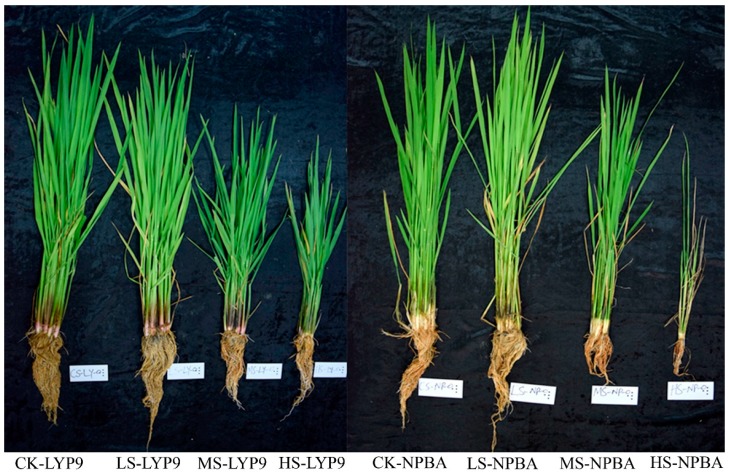
Effects of different levels of salt stress on the rice growth at the early stage in both LYP9 and NPBA.

Evaluations of cell membrane injury (CMI) in both LYP9 and NPBA rice revealed that salt concentrations and CMI are directly proportional, where higher salt concentrations cause severe cell membrane damage. The CMI was found to be higher in the HS condition as compared with MS, LS, and CK conditions in both rice cultivars (Figure 1). CMI was recorded as 5% for CK, 6.7% for LS, 7% for MS, and 15.2% for HS in LYP9. However, CMI in NPBA was recorded as 9.8% for CK, 10.6% for LS, and 11.9% for MS. Compared with the control (CK), the CMI in the LYP9 rice cultivar was increased by 34%, 40%, and 204% under LS, MS, and HS, respectively. On the other hand, CMI was increased by 8.1% (LS), and 21.4% (MS) in the NPBA rice, whilst rice seedlings died under HS conditions before reaching the maximum tillering stage in this genotype of rice (Figure 2). These results strongly suggest that salt stress negatively affects the cell membrane stability, and cell membrane integrity was found to be higher in LYP9 as compared with NPBA. Collectively, these results indicated that LYP9 is more tolerant to salt stress than NPBA.

### 2.4. Rice Root Activity

**Figure 3:**
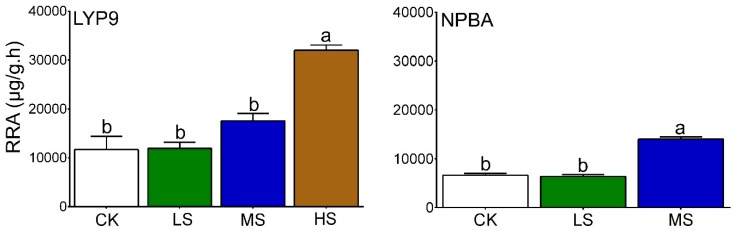
Rice root activity under different salt stress in LYP9 and NPBA. Bars denoted mean values ± SE (*n* = 3). Bars denoted mean values ±SE (*n* = 3). Values followed by different letters denote significant difference (*p* ≤ 05) according to LSD test. The similar lettering within rice genotype shows the significant and different lettering mean non-significance within treatment levels.

High root activity is an indicator of resistance against stress [31]. Rice root activity was increased by 2.1% for LS, 50.2% for MS, and 173.7% for HS as compared with CK in LYP9. In the case of NPBA, the rice root activity was decreased by 3.3% for LS, while it increased by 111.4% for MS, as compared to CK. In this study, the rice root activity was higher in LYP9 compared with NPBA under various salt stress levels, inferring the role of root activity in salt tolerance (Figure 3).

### 2.5. iTRAQ-Based Protein Identification at the Rice Maximum Tillering Stage

Quantitative proteomic analysis of three leaf samples (CK, LS, and MS) from NPBA rice and four leaf samples (CK, LS, MS, and HS) from LYP9 rice were performed using the iTRAQ method. In total, 5340 proteins were identified with 1% FDR (Table 2). In LYP9, 28, 368, and 491 proteins were found to be up-regulated under LS, MS, and HS treatments, respectively, as compared with the CK treatment. On the other hand, in NPBA, 239 and 337 up-regulated proteins were detected under the LS and MS treatments as compared with the CK treatment (Table 3). The longest length of enriched peptides was 7 to 18, with the mass error below 0.025 to 1.00 and with a high performing Pearson correlation coefficient with repeated samples, showing a high quality of the mass spectroscopy data and sample preparation. Proteins with a 1.2 fold change and Q-value of >0.05 were considered as differentially expressed proteins.

### 2.6. Identification of Differential Expressive Proteins in LYP9 and NPBA Subjected to Different Salt Stress Levels

**Figure 4:**
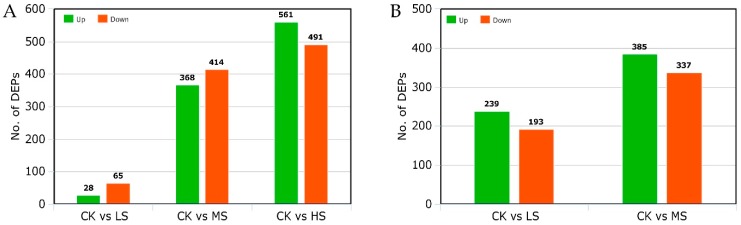
Identification of the differential expressive proteins (DEPs). (**A**) DEPs in LYP9 rice under various salt stress levels as compared with the control plants. (**B**) DEPs in NPBA under various salt stress levels as compared with the control plants. CK: control, LS: low salt, MS: medium salt, HS: high salt.

From the iTRAQ-based identified proteins in both rice genotypes, the proteins that showed a relative abundance of >1.2 fold or <0.8 fold in the salt stressed plants, as compared to the control, were considered to be differential expressive proteins (DEPs). In LYP9 rice, 1927 DEPs were identified under various salt levels. For instance, 93 (28 up-regulated, 65 down-regulated) DEPs were identified in the LS condition, 782 (368 up-regulated, 414 down-regulated) DEPs were identified in the MS condition, and 1052 (561 up-regulated, 491 down-regulated) DEPs were identified in the HS plants, as compared to the control (Figure 4A). On the other hand, 1154 DEPs were identified in the NPBA rice under the applied salt stress levels. Briefly, 432 (239 up-regulated, 193 down-regulated) DEPs were identified in the LS condition and 722 (385 up-regulated, 337 down-regulated) DEPs were identified in the MS plants, as compared with the control (Figure 4B). Identification of the DEPs in both rice genotypes indicated that, with an increase in the salt levels, the number of DEPs was also increased in both rice types. Further, under LS stress levels, the number of DEPs was significantly less in the salt tolerant LYP9 genotype than in the salt sensitive NBPA rice.

### 2.7. Gene Ontology (GO) and Kyoto Encyclopedia of Genes and Genomes (KEGG) Enrichment of the DEPs

**Figure 5:**
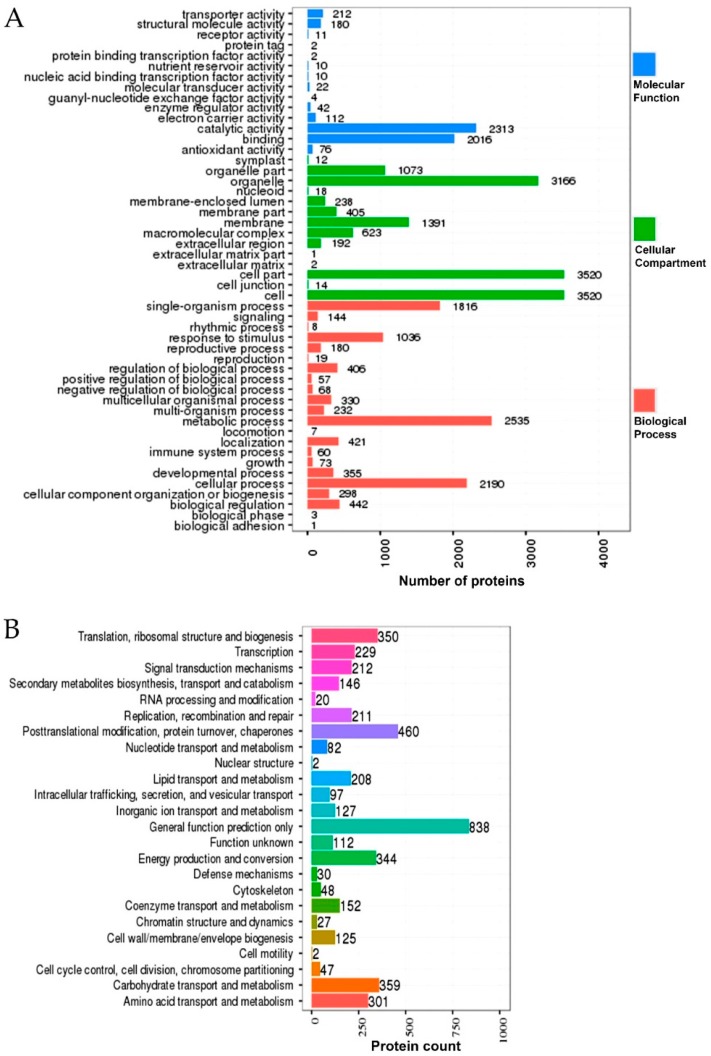
Gene ontology (GO) and Clusters of Orthologous Group (COG) analysis of the differentially responsive proteins in response to salt stress. (**A**) The distribution of number of differentially responsive proteins alongside their corresponding GO terms. Different colors represent different GO categories. (**B**) The distribution of number of differentially responsive proteins alongside their different functions as annotated by COG analysis.

To deduce the functionality and biological processes associated with the identified DEPs in the rice genotypes, GO analysis, Clusters of Orthologous Group (COG) annotations, and Kyoto Encyclopedia of Genes and Genomes (KEGG) enrichments were performed. The GO analysis revealed that the identified DEPs were associated with different molecular and biological processes (Figure 5A). Most of the identified DEPs in both rice genotypes were involved in cellular and metabolic processes (biological process). At the molecular level, most of the identified DEPs were involved in catalytic activity, binding, transporter and carrier activity, and structural molecule activity. Similarly, at the cellular component level, the identified DEPs were linked to the cell (membrane and cytoplasm) and organelles. In addition to that, COG analysis of the DEPs grouped them into 24 specific categories on the basis of their functional annotations (Figure 5B). Most of the DEPs were clustered in the “general functional prediction only” category, whereas the post-translational modifications, translation, energy production, carbohydrate metabolism, and amino acid metabolism clusters were found to be the other abundant ones. Altogether, these results suggest that, under salt stress in the rice, salt-responsive proteins might be involved in different metabolic and cellular processes and localize in different cell parts and organelles. 

**Figure 6:**
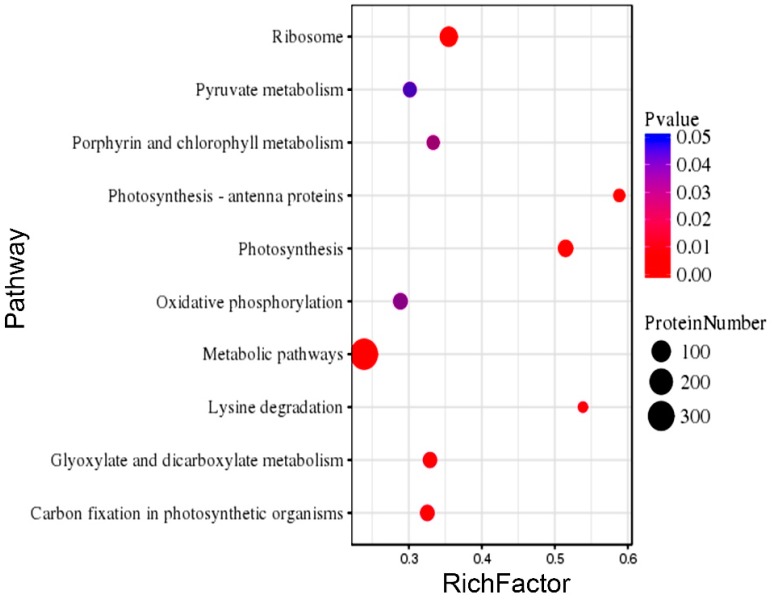
Top 10 pathway enrichments of the identified DEPs in LYP9 and NPBA by KEGG analysis. The corresponding pathways are listed on the Y-axis and the Rich factor values are mentioned along X-axis. Different sized dots represent the distribution of DEPs for a corresponding pathway, whereas, their color represents the *p* value.

**Figure 7:**
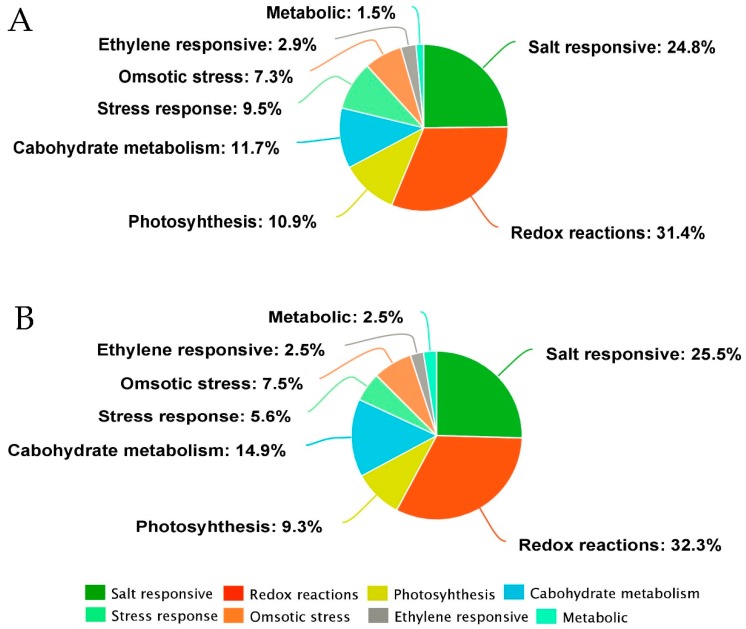
The major pathway annotations of the identified DEPs in LYP9 and NPBA rice. (**A**) Different pathways and their annotated DEP percentages in NPBA rice. (**B**) Different pathways and their annotated DEP percentages in LYP9.

**Figure 8:**
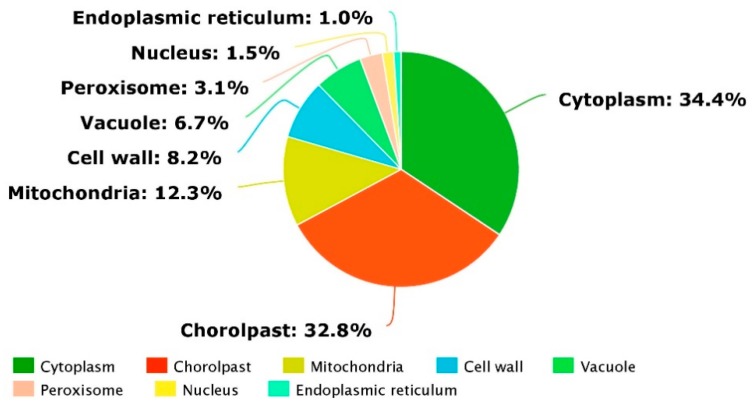
The predicted subcellular localization and compartmentation of the identified DEPs in LYP9 and NPBA.

In addition, the KEGG enrichment of the identified DEPs in both rice genotypes revealed their functionality as per the associated pathways. The KEGG pathways, including the metabolic pathway, oxidative phospohorylation, photosynthesis, lysine degradation, glyoxylate metabolism, carbon fixation, photosynthesis-antenna proteins, chlorophyll metabolism, pyruvate metabolism, and ribosomes were found to be the top 10 annotated pathways for the DEPs (Figure 6). From these, the metabolic pathways were found to be the primary enriched pathways in both the rice genotypes. Moreover, analysis of the detail of the KEGG enrichments and associated GO terms revealed that DEPs involved in the salt stress response, redox reactions, photosynthesis, and osmotic stress response were the most abundant in the rice genotypes (Figure 7). For instance, in LYP9, 41 salt-responsive proteins were found to be upregulated under various salt levels, whereas 26 upregulated DEPs were found in the NPBA rice. Similarly, 24 DEPs associated with carbohydrate metabolism were found to be upregulated in LYP9 rice, while 16 DEPs involved with carbohydrate metabolism were found to be upregulated in NPBA. The DEPs from both rice genotypes, with their corresponding fold changes as compared to the controls and their associated physiological pathways, are listed in Table 3. In addition, prediction of the subcellular localizations of the identified DEPs in both the rice genotypes revealed that most of the DEPs localize in the cytoplasm and chloroplasts (Figure 8).

## 3. Discussion

### 3.1. Biochemical Responses of Rice Plants to Salt Stress

Salt stress is a major concern in agriculture, affecting crop productivity across the world. Nutrient imbalance, due to the competition of Na^+^ and Cl^−^ with other nutrients, including potassium (K^+^), calcium (Ca^2+^), and nitrate (NO^3−^) ions, is a result of salt stress that compromises normal plant growth and development [8,9,10,11,12,32]. In addition, salt stress induces early leave-senescence and a decrease in photosynthesis area [33]. Moreover, osmotic imbalance, poor leaf growth, high CMI, and decreased root activity are associated with the typical salt stress responses in plants [31]. In the current study, the subjection of salt stress negatively affected rice growth in the early stages. All four levels of applied salt stress to both rice cultivars resulted in compromised growth parameters along with CMI. The degree of CMI was found to be higher in NPBA as compared with LYP9, suggesting LYP9 has a higher salt tolerance capacity than NPBA (Figure 1). Further, high rice root activity is usually associated with the interaction of the root with rhizosphere soil and the microbial environment [34], changes in physico-chemical status [35], and plant growth [36]. Further, by enhancing the root activity, plants cope better under an unfavorable environment [34] (Figure 3). In this study, the salt tolerance levels of LYP9 were found to be much higher than those of NPBA at high salt conditions (HS). LYP9 plants could survive by significantly increasing their root activities, whereas none of NPBA plants could survive at the same salt concentrations (Figure 2).

### 3.2. Proteomic Analysis in the Rice Genotypes Under Salt Stress

Both transcriptomic and proteomic dynamics occurring when subjected to salt stress have already been reported in several plants [37]. Further, the availability of substantial sequential information on rice has paved the way for the use of analytical proteomic studies, including iTRAQ analysis. In this study, iTRAQ-based protein identifications in LYP9 and NPBA cultivars revealed their proteome dynamics in response to salt stress. The comparative analysis of the total of identified proteins (5340) revealed that 93, 782, and 1052 proteins were differentially regulated in LYP9 as compared to the control (CK) under LS, MS, and HS salt stress conditions, respectively. On the other hand, in NPBA, 432 and 722 differentially expressed proteins were found as compared to CK under LS and MS salt stress conditions, respectively (Table 3). These results suggest that the numbers of identified proteins are in direct proportion to the increasing salt stress levels. In addition, the finding of increased numbers of differentially expressed proteins in between LS and MS in both cultivars, and in between LS and MS, and MS and HS in LYP9, further strengthens the proposed proportional relationship between differential protein expression and salt stress levels. Moreover, using the iTRAQ identified protein information, we compared the proteins expressed in LYP9 and NPBA, and thereby the biochemical pathways were identified, including salt stress-responsive protein synthesis, redox responses, photosynthesis, and other metabolic processes. Some of these pathways in response to salt stress have been confirmed in some of the previous studies [38,39]; therefore, the functions of the identified DEPs in this study are discussed further below.

**Figure 9:**
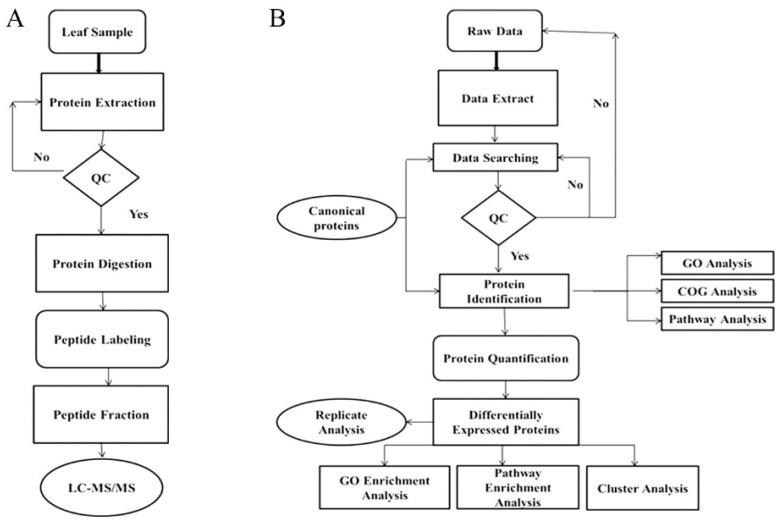
Schematic diagram of the experimental procedures and the complete pipeline for isobaric tags for relative and absolute quantitation (iTRAQ) bioinformatics quantification analysis. (**A**) Steps of the experiment of iTRAQ quantitative proteomics. (**B**) The bioinformatics analysis pipeline for the identified proteins from iTRAQ analysis. All the proteins (FDR < 0.01) proceeded with downstream analysis, including GO, COG, and Kyoto Encyclopedia of Genes and Genomes (KEGG).

The proteome dynamics and the DEPs in NPBA and LYP9 rice genotypes under different salt stress levels were determined by using iTRAQ analysis. Further, to detect and quantify the proteins in the rice genotypes, the high-resolution LC–MS/MS technique was employed. The identified proteins were quantified on automated software called IQuant [40]. Sequences of the identified DEPs were retrieved from the rice protein database based on the GI numbers, and a blastp algorithm was performed against the GO and KEGG databases. GO annotations of the DEPs were performed over three domains—cellular component, molecular function, and biological process—by using *R* software packages. Likewise, the COGs were delineated by using a PERL scripted pipeline. The pipeline of the iTRAQ-based protein identification and the subsequent bioinformatic characterizations are represented in Figure 9.

#### 3.2.1. Proteins Related to Salt Stress

The comparative proteomics study of both rice genotypes (LYP9 and NPBA) under salt stress revealed new insights into the salt resistance or sensitive mechanisms in rice. In both the rice genotypes, some of the major salt stress-responsive proteins exhibited differential up regulations as compared to the control, including malate dehydrogenase (gi|115482534), glucanase (gi|13249140), nascent polypeptide-associated complex (NAC) subunit (gi|115450217), methyltransferase (gi|115477769), and chloroplast inorganic pyrophosphatase (gi|46805452) (Table 3). Plant malate dehydrogenase (MDH) (EC 1.1.1.37) is a member of the oxidoreductase group that catalyzes the inter-conversion of malate and oxaloacetate in a redox reaction [24]. Further, MDH has been shown to play a vital role in regulating the salt stress response in plants [41,42]. Likewise, glucanase and inorganic pyrophosphatases have been associated with salt resistance properties in plants [43,44]. NAC has been reported to be involved in the translocation of newly synthesized proteins from the ribosomes to the endoplasmic reticulum during various physiological conditions, by directly interacting with the signal recognition particles. Further, overexpression of SaβNAC from *Spartina alterniflora* has been reported to enhance the salt tolerance in *Arabidopsis* [45]. In addition, methylation is often utilized by plants under unfavorable conditions as a strategy for gene regulation, protein sorting, and repairs [46]. IbSIMT1, a methyltransferase gene, has been observed to be activated by salt stress, and confers salinity resistance in sweet potato [47]. On the contrary, DEPs associated with salt stress responses, including glutathione peroxidase (GP) (gi|125540587), fructose-bisphosphate aldolase (FBA) (gi|218196772), pyruvate dehydrogenase (gi|125564321), and triosephosphate isomerase (TPI) (gi|125528336) were found to be significantly upregulated in LYP9, but down regulated in NPBA. Recently, the rice GP gene (*OsGPX3*) has been reported to play a vital role in regulating the salt stress response [48]. Rice plants with silenced *OsGPX3* were found to be highly salt sensitive, confirming the positive role of GP in salinity tolerance. FBA is involved in plant glucose pathways, including glycolysis and gluconeogenesis, and also plays a role in the Calvin cycle [49]. However, the FBA gene has been reported to exhibit induced expressions under salt stress in plants, indicating its role in salt stress. The FBA genes in *Arabidopsis* and *Camellia oleifera* were found to be strongly upregulated under salt stress, conferring salinity tolerance [48,50]. Likewise, the transcription of TPI genes has been reported to become active in rice in response to salt stress [51,52]. The upregulated expression of these salt related proteins in the salt-tolerant genotype LYP9, and their down regulation in the salt-sensitive NPBA, suggests that these genotypes possess a different protein pool in response to salinity. Moreover, the difference in salt tolerance between these two rice genotypes might have resulted due to the differential expression of these key proteins. A functional validation study, such as the Western blot or protein interactions, will add further insights to this hypothesis. 

#### 3.2.2. Proteins Related to Redox Reactions

Salt stress in plants induces osmotic imbalances, disrupts ion-homeostasis, and triggers oxidative damage, including the generation of reactive oxygen species (ROS) [53,54]. A fitting response to these adversities caused by salinity stress includes physiological and developmental changes, reprograming of salt-induced gene or proteins, and activation of ROS scavenging pathways [55]. In the current study, the proteomic analysis of LYP9 and NBPA revealed that redox reactions and ROS signaling are involved in the salt stress response in rice. Major enzymes involved in ROS signaling and redox reactions, including peroxidases (POD) (gi|125525683), superoxide dismutase (SOD) (gi|125604340), and glutathione s-transferase (GST) (gi|115459582), were found to be highly expressive in LYP9 and NPBA genotypes under the multiple salt stress levels we investigated (Table 3). Under salt stress, the cell membrane-bound peroxidases like NADPH oxidase and the diamine oxidases present in apoplast are activated, leading to generation of ROS [56,57]. In addition, SOD act as the first line of antioxidant defense in plants under multiple stress responses, and confer enhanced tolerance levels to oxidative stress [54]. Similarly, increased levels of GSTs in response to multiple stimuli have been reported in plants to mitigate oxidative stress [58]. Induced expressions and differential regulation of antioxidant enzymes, including PODs, SODs, and GSTs, have been reported by several studies in rice in response to salt stress [59,60]. Furthermore, comparative proteome analysis has confirmed the involvement of ROS and redox related protein in salt stress in plants, including alfalfa [61], searocket [62], maize [63], barley [64], and wheat [65]. Moreover, as many as 56 DEPs annotated with redox reaction functions were identified in both the rice genotypes under the various salt stress levels, suggesting oxidation and reduction reactions might be the key biochemical changes taking place in rice under salinity.

#### 3.2.3. Proteins Related to Photosynthesis

Photosynthesis is a major physiological process accounting for sustainability and energy production in plants. However, salt stress has adverse effects on the plant photosynthesis process by causing a decrease in the leaf cellular CO_2_ levels [7,66]. Additionally, salinity affects the Rubisco activity, retards chlorophyll synthesis, and destabilizes photosynthetic electron transport [66]. The findings from our study revealed that salt stress in rice affects the expression of the proteins involved in the photosynthesis process. These proteins, including the thylakoid lumenal protein (gi|115477166), psbP domain-containing protein 6 (gi|115440559), psbP-like protein 1 (gi|38636895), ferredoxin-thioredoxin reductase (gi|115447507), photosystem I 9K protein (gi|218186547), photosystem II oxygen-evolving complex protein 2 (gi|164375543), and protochlorophyllide reductase B (gi|75248671), were found to be highly expressed under salt stress conditions (Table 3). Thylakoid luminal protein is required for the functioning of photosystem II (PspB), whereas ferredoxin reductase is a key enzyme that facilitates the conversion of ferredoxin to NADPH in the photosystem I (PSI) complex, and these are also affected by salt stress [67,68]. Moreover, the psbP proteins, thylakoid luminal proteins, and ferredoxin reductase have been reported to be differentially expressed under salt stress [68]. Likewise, differential expression of photosystem proteins was reported in tomatoes in response to salt stress [69]. Similarly, the differential protein expression of protochlorophyllide reductase between the salt stress-induced and control, and its effects on chlorophyll biosynthesis, has been reported in rice [70]. Usually, in salt sensitive plants, salinity causes the down-regulation of photosynthesis proteins, compromising plant sustainability [2,71]. However, the analysis of iTRAQ-based proteomics revealed that the proteins involved in photosynthesis were upregulated in both rice genotypes, which might have aided the rice types to withstand salinity pressures. 

#### 3.2.4. Proteins Related to Carbohydrate Metabolism

Apart from being the building blocks in plants, soluble carbohydrates act as osmolytes, and thereby participate in salt tolerance in plants [72]. Besides, the onset of salt stress affects the protein dynamics in plants, resulting in differential protein accumulations [73]. In this study, several carbohydrate metabolism related proteins, including xyloglucan endotransglycosylase/hydrolase protein (XTH) (gi|115475445), β-glucosidase (gi|115454825), and polygalacturonase (gi|115479865), were found to be upregulated in both rice genotypes under various salt stress levels. XTH is known as a cell wall-modifying enzyme, however it also plays a role in salinity resistance responses in plants (Table 3). For instance, the constitutive and heterologous expression of CaXTH3 resulted in increased salt tolerance levels in *Arabidopsis* and tomato plants [74,75]. Similarly, β-glucosidase is a key enzyme in the cellulose hydrolysis process, and has been reported to be involved in the salt stress response. In barley, the activity of an extracellular β-glucosidase was reported to be highly induced in response to salt stress, and cause abscisic acid-glucose conjugate hydrolysis [76]. Further, the overexpression of *Thkel1*, a fungal gene that modulates β-glucosidase activity, improved the salt tolerance levels in transgenic *Arabidopsis* plants [77]. Polygalacturonase, another enzyme capable of hydrolyzing the α-1,4 glycosidic bonds, participates in the salt stress responses in plants. Characterization of the salt stress responses and the associated signal transduction pathways in *Arabidopsis* revealed the elevated transcript accumulation of a polygalacturonase gene (*At1g48100*) under salt stress [78]. However, several proteins related to carbohydrate metabolism, including xylanase inhibitor protein (XIP) (gi|297605789, gi|115467998) and MDH (gi|116310134), were found to be downregulated in the NPBA rice, while being upregulated in the LYP9 rice. MDH is a key enzyme in stress responses and actively participate in the tricarboxylic acid (TCA) cycle [74]. In the current study, upregulated expression of MDH was found in LYP9, however down-regulation in NPBA suggests the inhibition of the TCA cycle in the salt sensitive NPBA, but not in the tolerant LYP9 genotype. Further, OsXIP was reported to be induced under various abiotic stresses, including salt stress, and to take part in the rice defense mechanisms against several biotic and abiotic stresses [79]. Moreover, the induced many-fold expression of the carbohydrate metabolism related proteins in LYP9, but their down regulation in NPBA, indicates that carbohydrate metabolism might be a major physiological process that is affected under salinity in rice, and can show the dynamic changes in protein expression depending on the salt tolerance capacity of a genotype.

#### 3.2.5. Proteins Related to Osmotic Stress

Often, salt stress induces the reduction of cellular water potential, causing osmotic stress to the plant. Osmotic stress responses in plants can be very complex in higher plants, including rice [80]. In this study, 11 osmotic stress related proteins were differentially expressed in both rice genotypes under various salt levels, suggesting salt stress in rice leads to the onset of osmotic stress. For instance, a putative lipid transferase protein (gi|297612544) identified as a DEP in both the rice genotypes was found to be upregulated under salt stress. The induced expression of *TSW12* and *SiLTP,* coding the lipid transferase proteins in tomato and foxtail millet plants, has been reported under salt stress [80,81]. Conversely, osmotic stress responsive proteins such as sucrose synthase (gi|125544232) and NADH dehydrogenase (gi|115473055) were found to exhibit an induced response in LYP9 rice under salt stress, but were not significantly induced in the NPBA rice. Sucrose synthase (Sus) is the major enzyme in sucrose metabolism, however it also plays a part in osmotic stress responses in plants. In *Arabidopsis*, up-regulation of Sus1 has been reported in response to osmotic stresses and water deficit conditions [82]. In addition, involvement of Sus in the osmotic stress response has been reported in Beta vulgaris [83]. On the other hand, NADH dehydrogenase facilitates electron transfer from NADH to the mitochondrial respiratory chain [84]. The up-regulation of NADH dehydrogenase under salt stress indicates an increase in the ATP pool in the LYP9 rice, subsequently aiding in sustainable plant growth and salinity tolerance. However, no induced expression of the same in NPBA suggests that, under salt stress, the ATP pool might decrease, resulting in declining plant growth (Table 3). 

#### 3.2.6. Proteins Related to Other Metabolic Processes

Salt stress alters the protein pool that contributes to many metabolic mechanisms, such as stress responses, energy metabolism, and phytohormone synthesis [23,85]. In this study, several DEPs have been identified in the rice genotypes under salt stress, with various physiological and metabolic functions. For instance, putative glucan endo-1,3-β-glucosidase 4 (gi|297607511) was found to be up-regulated in both rice types under salt stress conditions. Similar findings were reported in cotton plants, where the subjected salt stress caused an increased accumulation of glucan endo-1,3-β-glucosidase [23]. Further, the strong induced response of a putative zinc finger protein (gi|28971968) was found under salt stress in both rice genotypes. Induced expression of gene finger proteins has been associated with several stresses, including salt stress. Overexpression of a rice zinc-finger protein OsISAP1 in transgenic tobacco resulted in enhanced abiotic stress tolerance levels, including salinity, dehydration, and cold [86]. Recently, OsZFP213 was reported to interact with OsMPK3, conferring salinity tolerance in rice [87]. In addition, many other proteins with annotated functions or which are uncharacterized were found to be differentially regulated at various salt levels in the rice genotypes. Moreover, these results collectively suggest that salinity affects many physiological processes in rice, irrespective of their salt tolerance levels. Furthermore, the protein pool of a salt tolerant and a salt sensitive rice genotype might differ at a specific point of time, which could be the basic reason of their differential salt tolerance responses (Table 3).

## 4. Materials and Methods

### 4.1. Plant Material and Growth Conditions

A pot culture experiment was conducted in a greenhouse at China National Rice Research Institute (39°4′49′′ N, 119°56′11′′ E), Zhejiang Province, China, during the rice growing season (May–November, 2017). Two rice cultivars (origin, China and Japan), Liangyoupiejiu (LYP9, Hybrid, *indica*) and Nipponbare (NPBA, *japonica*) were used as the planting materials. Thirty-day old seedlings were transplanted in pots (45 × 30 cm) with different salt stress levels and 23 kg air-dried soil. The experimental soil was loamy clay with an average bulk density of 1.12 g/cm, 4.7% organic matter, 0.0864 dS/m EC, and 5.95 pH. Each pot contained six rice seedlings with three replications.

Sodium chloride (NaCl) was used in each pot to develop artificial salinity in soil until the maximum tillering stage of the rice seeding was reached (about 45 days). The treatments were comprised of four NaCl levels: 0 (control, CK), 1.5 g NaCl/kg dry soil (low salt stress, LS), 4.5 g NaCl/kg dry soil (moderate salt stress, MS), and 7.5 g NaCl/kg dry soil (high salt stress, HS). After salinity development, the corresponding EC for these levels was 0.086 dS/m (CK), 1.089 dS/m (LS), 3.20 dS/m (MS), and 4.64 dS/m (HS). 

Nitrogen was applied in the form of urea (N: 46%), phosphorous as superphosphate (P_2_O_5_: 12%), and potassium as potassium sulfate (K_2_O: 54%). Urea was used at the rate of 4.02 g/pot in two splits: 50% was applied as the basal dose, and 50% was applied at the tillering stage. Potassium sulfate (3.08 g/pot) was applied in two equal splits, as a basal dose and at the tillering stage, while the whole amount of superphosphate (6.93 g/pot) was applied as a basal dose.

### 4.2. Soil and Plant Sampling

Rice flag leaves were collected at the maximum tillering stage and stored at −80 °C after being frozen in liquid nitrogen. Plants were collected for measurement of Na^+^ and Cl^−^ contents in the roots and leaves at the maximum tillering stage. Soil samples were collected at the transplanting stage and at the maximum tillering stage to check the Na^+^ and Cl^−^ contents in the soil. Five flag leaves with three replicates were collected to measure the cell membrane injury in rice leaves at the maximum tillering stage, while root samples were collected to measure the rice root activity. All these experiments were performed with three independent biological replicates.

### 4.3. Leaf Proteomics Analysis Pipeline

#### 4.3.1. Protein Extraction

A total of 1–2 g of plant leaves with 10% PVPP were ground in liquid nitrogen and then sonicated on ice for 5 min in Lysis buffer 3 (8M Urea and 40 mM Tris-HCl containing 1 mM PMSF, 2 mM EDTA, 10 mM DTT, and pH 8.5) with 5 mL of samples. After centrifugation, 5 mL of 10% TCA/acetone with 10 mM DTT were added to the supernatant to precipitate the proteins. The precipitation step was repeated with acetone alone until the supernatant became colorless. The proteins were air dried and re-suspended in Lysis buffer 3. Ultra-sonication on ice for 5 min was used to improve protein dissolution with the help of Lysis buffer 3. After centrifugation, the supernatant was incubated at 56 °C for 1 h for reduction, and then alkylated by 55 mM iodoacetamide (IAM) in the dark at room temperature for 45 min. Acetone (5 mL) were used to precipitate the proteins and stored at –80 °C. The quality and quantity of the isolated proteins were estimated by performing Bradford assay and SDS-PAGE [88].

#### 4.3.2. Digestion of Proteins and Peptide Labeling

About 100 µg of the protein solution with 8 M urea was diluted four times with 100 mM TEAB. For the digestion of the proteins, Trypsin Gold (Promega, Madison, WI, USA) was used at a ratio of trypsin: protein of 40:1, at 37 °C, and was put into the samples overnight. After the digestion with trypsin, Strata X C18 column (Phenomenex, Torrance, CA, USA) were used to desalt the peptides and vacuum-dry them according to the manufacturer’s protocol. For peptide labeling, the peptides were dissolved in 30 µL 0.5 M TEAB. Then, the peptide labeling was performed by an iTRAQ reagent 8-plex kit. The labeled peptides with different reagents were combined and desalted with a Strata X C18 column (Phenomenex), and vacuum-dried.

#### 4.3.3. Peptide Fractionation and HPLC

The peptide fractionations were performed by using a Shimadzu LC-20AB HPLC pump attached to a high pH RP column. About 2 mL of the reassembled peptides with buffer A (5% ACN, 95% H_2_O, pH 9.8) was loaded on a 5 μm particulate column (Phenomenex). The flow rate was adjusted to 1 mL/min with a 5% buffer B (5% H_2_O, 95% ACN, pH 9.8) gradient for 10 min, with 5–35% buffer B for 40 min, and with 35–95% buffer B for 1 min, to separate the peptides. An incubation of 3 min in 95% buffer B, and for 1 min in 5% buffer B, followed this, before the final equilibration with 5% buffer B. Each peptide fraction was collected at 1 min time intervals, and OD of the eluted fractions were measured at 214 nm. Twenty fractions were pooled together and vacuum dried. Post drying, the fractions were re-suspended in buffer A solution (2% CAN; 0.1% FA in water) individually and centrifuged. Then, the supernatant was collected and loaded onto a C18 trap column with a rate of 5 μL/min by using a LC-20AD nano-HPLC device (Shimadzu, Kyoto, Japan). Peptide elutions were performed afterwards and separated by using an analytical C18 column with an inner diameter of 75 μm. The gradients were run at 300 nL/min starting from 8 to 35% of buffer B (2% H_2_O; 0.1% FA in ACN) for 35 min, with an increase up to 60% in 5 min, then were maintained at 80% buffer B for 5 min before returning to 5% in 6 s, with a final equilibration period of 10 min.

#### 4.3.4. Mass Spectrometer Detection

The spectrometric data were acquired using a TripleTOF 5600 System (SCIEX, Framingham, MA, USA) fit to a Nano-Spray III source (SCIEX, Framingham, MA, USA) and a pulled quartz tip-type emitter (New Objectives, Woburn, MA, USA), which was controlled with the franchise software Analyst v1.6 (AB-SCIEX, Concord, ON, Canada). The MS data procurements were undertaken as per the following conditions: the ion spray voltage was set to 2300 V, the curtain gas was set to 30, the nebulizer gas was set to 15, and the interface heater temperature was 150 °C. High sensitivity mode was used for the whole data acquisition process. The MS1 accumulation time was set to 250 ms, while 350–1500 Da was the allowed mass range. At least 30 product ion scans were collected based on the MS1 survey intensity, exceeding a threshold of 120 counts/s and a 2^+^ to 5^+^ charge-state. A value of ½ peak width was set for the dynamic exclusion. The collision energy was adjusted to all precursor ions for the collision-induced dissociation for the iTRAQ data acquisition, and the Q2 transmission window for 100 Da was at 100%. Three independent biological replicates were included for each sample in the experiment.

### 4.4. Cell Membrane Injury

Cell membrane injury (CMI) was determined by using flag leaves. Twenty pieces (1 cm diameter) were cut from these flag leaves, and were submerged into 20 mL distilled water (DI) contained in test tubes. The test tubes were kept at 10 °C in an incubator for 24 h. After 24 h, the samples were kept at 25 °C to warm the samples, and the electrical conductivity (C1) of the samples was measured. These samples were then autoclaved for 20 min at 120 °C and the electrical conductivity (C2) was determined again. Cellular injury was determined by using the following formula [89]:

(1) $$Cellmembraneinjury=\frac{C1}{C2}\times 100$$

where, ‘C’ refers to EC 1 and 2. The experiment was performed with three independent biological replicates.

### 4.5. Rice Root Activity

Rice root activity was analyzed by the triphenyl tetrazolium chloride (TTC) method [90]. Briefly, rice root samples (0.5 g, root tips) were taken, and 5 mL of the phosphate buffer (pH 7) and 5 mL 0.4% TTC (Vitastain, C_19_H_15_N_4_Cl) were added to keep the root activity alive. The samples were kept in an incubator in the dark at 37 °C for 3 h. After 3 h, the samples were taken out and 1 mL 1 mol/L H_2_SO_4_ was added to stop the reaction. The rice roots were then removed from the test tubes. These root samples were ground by adding a pinch of silica sand, and mixed with 8 mL ethyle acetate. The extract was transferred to test tubes and a 10 mL final volume was reached by adding ethylene acetate. These samples were analyzed using a spectrophotometer (UV-2600, UV-VIS Spectrophotometer Shimadzu) at 485 nm. The formula used for calculation of root activity is as follows:

(2) $$RootActivty=\frac{C}{W/3}$$

where C is the concentration of the samples calculated from a standard curve. W is the weight of the root samples. The experiment was performed with three independent biological replicates.

### 4.6. Na^+^ Concentration in the Soil and Plants

Na^+^ was extracted from the soil by ammonium acetate solution using Rihards (1954) method [91]. About 5 g ground (particle size ≤ 2mm) air dried soil was placed in 250 mL plastic bottles and 50 mL ammonium acetate (NH_4_OAc, 1 mol/L) was added. These bottles were kept on a shaker for 30 min at 120 rpm. After that, the samples were filtered by using filter paper to obtain the soil solution. 

Na^+^ in the plants’ parts was extracted by digestion with sulfuric acid (H_2_SO_4_) by following Rihards (1954) method [91] with the necessary modifications. About 0.3 g ground (particle size ≤ 2 mm) root and leaf were taken in 50 mL glass tubes and mixed with 5 mL H_2_SO_4_. These glass bottles were kept overnight. The samples were put into the fume hood and were incubated at 320 °C for 2 h. After 2 h, hydrogen peroxide solution (H_2_O_2_) was added drop by drop and the samples were mixed until a whitish or transparent color appeared. Then, the samples were cooled at room temperature before being filtered by using filter paper to get the plant part extracts. 

The soil and plant extracts were used to measure the sodium ions (Na^+^) by using a flame photometer. The standards used were 0, 2, 4, 6, 8, 10, 15, and 20 mL NaCl. The final soluble sodium (Na) in soil was measured by using the formula:

(3) $$\mathrm{Na}\left(\frac{\mu g}{g}\right)=\frac{A\times C}{W}$$

where A is the total volume of the extract (mL), C is the sodium concentration values given by the flame-photometer (µg/mL), and W is the weight of the air dried soil (g). The experiment was performed with three independent biological replicates.

### 4.7. Cl^−^ Concentration in the Soil and Plants

About 10 g air dried soil (particle size ≤ 2mm) was placed in 250 mL plastic bottles and mixed with 50 mL deionized water. These bottles were transferred onto a shaker and were shaken for 5 min at 180 rpm. The samples were then filtered by using filter paper to obtain the soil solution extract for Cl^−^.

Plant samples weighing approximately 0.1 g were placed in 50 mL glass tubes and mixed with 15 mL deionized water. The tubes were transferred into a hot water bath and kept for 1.5 h. The samples were then diluted with 25 mL deionized water after cooling at room temperature.

The soil and plant extracts were used to measure the chloride (Cl^−^) by using a chloride assay kit (QuantiChrom^TM^ Chloride Assay Kit, 3191 Corporate Place Hayward, CA 94545, USA) following the manufacturer’s instructions. The standards used were 0, 10, 20, 30, 40, 60, 80, and 100 mL. The final chloride concentration in the solution was measured by the formula:

(4) $$\mathrm{Chloride}=\frac{\mathrm{ODsample}-\mathrm{ODblank}}{\mathrm{Slop}}\times n\left(\frac{\mathrm{mg}}{\mathrm{dL}}\right)$$

where ODsample is the OD 610 nm values of the samples, and ODblank is the OD 610 nm values of the blanks (water). The experiment was performed with three independent biological replicates.

### 4.8. Statistical Analysis

The statistical software package IBS SPSS Statistics 19.0 was used for the analyses of data. For evaluating the statistical significance of the biochemical parameters, a one-way ANOVA was employed with LSD at the level of *p* = 0.05. For the iTRAQ-based protein quantification, all identified DEPs were required to satisfy the *t*-test at *p* ≤ 0.05, and with a fold change ratio of >1.2 or <0.8.

## 5. Conclusions

Using comparative iTRAQ-based protein quantification, the proteome dynamics of LYP9 and NPBA rice were explored in this study. The results from the study suggest that rice cell membrane integrity was inversely correlated and root activity was positively correlated with the concentration of salinity. Furthermore, the physiological processes, including carbohydrate metabolism, redox reactions, and photosynthesis, made significant contributions towards the salt tolerance in rice. The number of differentially expressed proteins—salt responsive proteins in particular—suggested that the protein pool in response to salt stress is different in a salt tolerant compared to a susceptible rice genotype. Finally, the *indica* rice LYP9 showed promising results under the subjected salt stress levels, and can be selected over the *japonica* NPBA for salt tolerance. Further works deciphering the functions of some particular proteins of interest will add new insights into their roles in salt tolerance in rice.

## Figures and Tables

**Table 1:** Differential Na^+^ and Cl^−^ uptake from soil to leaf via root in LYP9 and NPBA under different salt stress levels at rice maximum tillering stage.

Cultivars	Treatments	Na^+^ (mg/g)		Cl^−^ (mg/g)		Na^+^ (mg/g)	Cl^−^ (mg/g)
		Root	Leaf	Root	Leaf	Soil	Soil
**LYP9**	**CK**	0.7 ± 0.05*d*	0.2 ± 0.03*c*	0.5 ± 0.3*d*	6.8 ± 0.4*d*	0.2 ± 0.01*d*	0.04 ± 0.01*e*
	**LS**	1.1 ± 0.03*bc*	0.5 ± 0.08*b*	1.6 ± 0.7*cd*	12.4 ± 1.7*bcd*	1.0 ± 0.03*c*	0.6 ± 0.06*de*
	**MS**	1.5 ± 0.04*b*	0.8 ± 0.10*a*	7.9 ± 1.3*ab*	17.6 ± 2.4*ab*	1.7 ± 0.04*b*	2.2 ± 0.06*bc*
	**HS**	1.6 ± 0.09*a*	0.9 ± 0.11*a*	9.5 ± 1.6*a*	19.1 ± 2.9*a*	2.0 ± 0.07*a*	2.4 ± 0.33*b*
**NPBA**	**CK**	0.7 ± 0.03*d*	0.15 ± 0.01*cd*	1.0 ± 0.3*d*	9.9 ± 0.9*cd*	0.2 ± 0.01*d*	0.01 ± 0.01*e*
	**LS**	1.0 ± 0.07*c*	0.3 ± 0.01*c*	3.8 ± 0.4*c*	14.7 ± 2.8*abc*	1.0 ± 0.02*c*	0.7 ± 0.08*e*
	**MS**	1.3 ± 0.07*b*	0.9 ± 0.02*a*	6.3 ± 0.6*b*	18.7 ± 2.4*ab*	1.6 ± 0.12*b*	1.6 ± 0.035*d*
	**HS**	-	-	-	-	2.2 ± 0.07*a*	3.0 ± 0.23*a*

Values are denoted as mean ± SE (*n* = 3). Values followed by different letters denote significant difference (*p* ≤ 0.05) according to LSD test. Abbreviations: control (no salt stress, CK), low salt stress (LS), moderate salt stress (MS), and high salt stress (HS), Liangyoupeijiu (LYP9), Nipponbare (NPBA). The similar lettering within rice genotype shows the significant and different lettering mean non-significance within treatment levels.

**Table 2:** Overview of the total protein identification in both rice genotypes.

Total Spectra	Spectra	Unique Spectra	Peptides	Unique Peptide
402,823	71,146	53,833	21,741	18,899

**Table 3:** Differentially expressed proteins in NPBA and LYP9 rice under different salt levels with 1.2 fold change and Q-value > 0.05.

Protein ID	NCBI Accession	Protein Name	NPBA		LYP9		
			LS vs. CK	MS vs. CK	LS vs. CK	MS vs. CK	HS vs. CK
**Salt responsive**							
tr|B9FWE4|B9FWE4_ORYSJ	gi|222636749	Uncharacterized protein	1.516	1.415	0.906	1.234	1.255
tr|A2Y7R4|A2Y7R4_ORYSI	gi|115465579	Malate dehydrogenase	1.393	2	1.014	1.488	1.573
tr|B8BBS3|B8BBS3_ORYSI	gi|115476908	Os08g0478200 protein	1.389	1.593	0.951	1.402	2.706
tr|A2WT84|A2WT84_ORYSI	gi|115438875	Malate dehydrogenase	1.897	2.835	1.027	1.871	2.006
tr|A0A0P0VS15|A0A0P0VS15_ORYSJ	gi|115450217	Nascent polypeptide-associated complex subunit β (Fragment)	2.523	2.558	1.017	1.594	1.384
tr|A2XA10|A2XA10_ORYSI	gi|46805452	Os02g0768600 protein	1.506	2.225	1.071	2.213	2.212
tr|A0A190X658|A0A190X658_ORYSI	gi|115477769	l-isoaspartate methyltransferase	1.575	2.403	0.901	1.591	1.69
sp|Q43008|SODM_ORYSJ	gi|115463191	Superoxide dismutase	1.775	2.06	1.071	1.534	1.828
sp|Q9FE01|APX2_ORYSJ	gi|115474285	Ascorbate peroxidase	1.308	1.26	0.966	1.227	1.119
sp|Q07661|NDK1_ORYSJ	gi|61679782	Nucleoside diphosphate kinase 1	1.295	1.816	0.909	1.068	1.435
sp|Q5N725|ALFC3_ORYSJ	gi|297598143	Fructose-bisphosphate aldolase 3	1.399	1.639	1.023	1.089	1.532
sp|Q7XDC8|MDHC_ORYSJ	gi|115482534	Malate dehydrogenase	1.37	1.749	1.004	1.284	1.523
tr|A2X753|A2X753_ORYSI	gi|115447273	Os02g0612900 protein	1.441	1.552	1.036	1.506	1.597
tr|A2X7X9|A2X7X9_ORYSI	gi|125540544	Putative uncharacterized protein	1.152	1.502	0.882	1.378	1.25
tr|A0A0P0VTX8|A0A0P0VTX8_ORYSJ	gi|108706531	Os03g0182600 protein	0.852	1.876	0.908	0.949	1.367
tr|E0X6V4|E0X6V4_ORYSJ	gi|306415973	Triosephosphate isomerase	1.003	1.232	1.027	1.107	1.256
tr|A2ZAA7|A2ZAA7_ORYSI	gi|115483468	Nucleoside diphosphate kinase	1.197	2.406	0.882	1.08	1.768
tr|Q9ATR3|Q9ATR3_ORYSA	gi|13249140	Glucanase	1.063	2.009	0.876	0.912	1.39
tr|A2ZIH2|A2ZIH2_ORYSI	gi|115487556	Expressed protein	1.048	1.561	0.957	1.204	1.424
tr|B9FV80|B9FV80_ORYSJ	gi|222636335	Peroxidase	0.888	1.812	0.966	1.298	1.814
tr|B8B893|B8B893_ORYSI	gi|218199240	Plasma membrane ATPase	1.404	1.435	0.906	0.716	0.86
tr|A2XA20|A2XA20_ORYSI	gi|115448935	Proteasome subunit β type	0.862	1.109	0.946	1.067	1.059
tr|A2Y628|A2Y628_ORYSI	gi|125552829	Cysteine proteinase inhibitor	0.96	1.775	1.056	1.438	2.205
tr|Q9ZNZ1|Q9ZNZ1_ORYSA	gi|4097938	Beta-1,3-glucanase	0.795	1.711	1.003	0.932	1.619
tr|A2ZCK1|A2ZCK1_ORYSI	gi|148762354	Alcohol dehydrogenase 2	0.63	0.866	1.055	1.019	1.019
sp|A2XFC7|APX1_ORYSI	gi|158512874	l-ascorbate peroxidase 1	1.216	1.343	0.942	1.21	1.327
tr|A2X822|A2X822_ORYSI	gi|125540587	Glutathione peroxidase	0.717	0.617	0.924	1.77	1.567
tr|A2XFD1|A2XFD1_ORYSI	gi|125543402	Putative uncharacterized protein	1.1	1.543	0.943	1.23	1.554
tr|A2YLI3|A2YLI3_ORYSI	gi|115472191	Os07g0495200 protein	1.159	1.821	0.971	1.505	1.818
tr|B8ADI1|B8ADI1_ORYSI	gi|218187601	NADH-cytochrome b5 reductase	0.771	0.72	1.081	2.182	2.316
tr|A2YSB2|A2YSB2_ORYSI	gi|115475275	Os08g0205400 protein	1.587	2.217	0.908	1.597	1.194
tr|B8AY35|B8AY35_ORYSI	gi|218196772	Fructose-bisphosphate aldolase	0.458	0.214	0.964	0.74	1.505
tr|B8AY17|B8AY17_ORYSI	gi|218196757	Putative uncharacterized protein	0.725	0.849	0.996	1.174	1.649
tr|Q9ZNZ1|Q9ZNZ1_ORYSA	gi|4097938	Beta-1,3-glucanase	0.795	1.711	1.003	0.932	1.619
sp|Q941Z0|NQR1_ORYSJ	gi|115442299	Putative uncharacterized protein	0.686	0.766	0.984	0.931	1.369
tr|A2WWV4|A2WWV4_ORYSI	gi|125528336	Putative uncharacterized protein	0.518	0.55	1.031	1.159	1.304
sp|P93438|METK2_ORYSJ	gi|3024122	*S*-adenosylmethionine synthase	1.282	1.017	1.013	1.226	1.092
tr|A2XUB9|A2XUB9_ORY I	gi|90265194	B0812A04.3 protein	1.074	1.225	1.215	1.186	1.437
tr|A2Z2Z0|A2Z2Z0_ORYSI	gi|125564321	Putative uncharacterized protein	1.01	0.776	0.921	1.202	1.102
tr|B8AEU4|B8AEU4_ORYSI	gi|218191814	Putative uncharacterized protein	0.954	1.212	0.948	0.908	1.134
tr|A0A0P0VTX8|A0A0P0VTX8_ORYSJ	gi|108706531	Os03g0182600 protein	0.852	1.876	0.908	0.949	1.367
tr|Q688M9|Q688M9_ORYSJ	gi|51854423	putative endo-1,31,4-β-D-glucanase	1.16	1.14	0.992	1.111	1.144
tr|B8ATW7|B8ATW7_ORYSI	gi|115460338	Os04g0602100 protein	1.386	1.494	1.115	1.448	1.482
sp|Q7FAH2|G3PC2_ORYSJ	gi|115459078	Glyceraldehyde-3-phosphate dehydrogenase 2	0.887	1.03	1.004	0.996	1.196
tr|Q0JG30|Q0JG30_ORYSJ	gi|297598314	Os01g0946500 protein	0.95	0.844	0.995	0.799	0.959
tr|Q6L5I4|Q6L5I4_ORYSJ	gi|47900421	Putative aldehyde dehydrogenase	0.735	0.737	0.911	1.167	1.008
sp|A2XW22|DHE2_ORYSI	gi|81686712	Glutamate dehydrogenase 2	1.177	1.142	0.912	0.8	1.184
sp|Q7FAY6|RGP2_ORYSJ	gi|115461086	Amylogenin	1.357	1.021	0.776	0.558	0.683
sp|Q259G4|PMM_ORYSI	gi|115461390	Phosphomannomutase	0.836	1.19	1.007	0.924	1.216
**Photosynthesis related**							
tr|A2YWS7|A2YWS7_ORYSI	gi|115477166	Os08g0504500 protein	1.317	2.22	0.883	1.852	1.77
tr|Q2QWM7|Q2QWM7_ORYSJ	gi|108862278	Os12g0190200 protein	1.053	1.535	0.91	1.363	1.279
tr|B8BCC6|B8BCC6_ORYSI	gi|115477246	Os08g0512500 protein	2.311	3.409	1.022	1.551	1.349
tr|A2XZK1|A2XZK1_ORYSI	gi|125550552	Putative uncharacterized protein	1.246	1.015	1.301	2.61	2.439
tr|B8AAX3|B8AAX3_ORYSI	gi|115440559	Os01g0805300 protein	1.418	2.178	0.986	1.943	1.703
tr|Q0D6V8|Q0D6V8_ORYSJ	gi|297607127	Os07g0435300 protein	2.246	3.387	0.982	2.305	2.053
tr|Q7XHS1|Q7XHS1_ORYSJ	gi|115472141	2Fe-2S iron-sulfur cluster protein-like	1.016	1.414	0.936	1.62	1.548
tr|A2X7M2|A2X7M2_ORYSI	gi|115447507	Os02g0638300 protein	1.096	1.611	1.14	1.73	1.825
tr|B0FFP0|B0FFP0_ORYSJ	gi|115470529	Chloroplast 23 kDa polypeptide of PS II (Fragment)	1.319	1.705	0.997	1.747	1.609
tr|Q7M1U9|Q7M1U9_ORYSA	gi|218186547	Photosystem I 9K protein	1.832	3.172	1.027	2.206	2.383
tr|A0A0P0XF80|A0A0P0XF80_ORYSJ	gi|38636895	Os08g0347500 protein	1.642	2.347	0.926	1.756	1.81
tr|Q7M1Y7|Q7M1Y7_ORYSA	gi|164375543	Photosystem II oxygen-evolving complex protein 2 (Fragment)	1.77	2.373	0.989	2.015	1.756
tr|B8AJX7|B8AJX7_ORYSI	gi|115455221	Serine hydroxymethyltransferase	2.24	2.885	1.078	1.525	1.334
tr|B8AY24|B8AY24_ORYSI	gi|218196765	Putative uncharacterized protein	1.288	1.56	1.026	1.639	1.326
sp|Q6Z2T6|CHLP_ORYSJ	gi|297599916	Geranylgeranyl reductase	0.956	1.173	0.973	0.957	1.174
sp|P0C420|PSBH_ORYSA	gi|11466818	Photosystem II reaction center protein H	0.795	0.694	1.029	0.864	1.14
**Oxidation reduction responsive**							
tr|A3BVS6|A3BVS6_ORYSJ	gi|125604340	Superoxide dismutase	1.512	1.903	0.96	1.618	1.684
sp|Q6H7E4|TRXM1_ORYSJ	gi|115447527	Putative uncharacterized protein	0.941	1.681	1.049	1.75	2.489
sp|Q9SDD6|PRX2F_ORYSJ	gi|115435844	Peroxiredoxin-2F, mitochondrial	1.363	1.772	1.021	1.642	1.687
tr|B7FAE9|B7FAE9_ORYSJ	gi|215769368	Glutathione peroxidase	0.98	1.347	0.965	1.38	1.176
tr|A2Y043|A2Y043_ORYSI	gi|125550744	Peroxidase	1.232	2.046	0.87	0.633	1.271
tr|Q9FTN6|Q9FTN6_ORYSJ	gi|115434034	Os01g0106300 protein	0.732	1.977	0.788	0.606	1.476
tr|A2X2T0|A2X2T0_ORYSI	gi|55700921	Peroxidase	0.775	1.122	0.913	0.85	1.697
tr|O22440|O22440_ORYSA	gi|115474063	Peroxidase	1.763	2.554	0.963	2.051	1.612
tr|A3A7Y3|A3A7Y3_ORYSJ	gi|125582491	Uncharacterized protein	1.099	1.361	1.101	1.555	2.4
tr|B9FL20|B9FL20_ORYSJ	gi|115464801	Uncharacterized protein	1.175	1.416	0.965	1.159	1.356
tr|Q9AS12|Q9AS12_ORYSJ	gi|115436300	Peroxidase	4.654	5.188	0.78	1.948	2.334
tr|B8ATW7|B8ATW7_ORYSI	gi|115460338	Os04g0602100 protein	1.386	1.494	1.115	1.448	1.482
tr|B9FCM4|B9FCM4_ORYSJ	gi|116309795	OSIGBa0148A10.12 protein	2.208	2.05	1.017	1.365	1.123
tr|Q0JB49|Q0JB49_ORYSJ	gi|115459848	Glutathione peroxidase	1.449	1.435	0.933	1.537	1.271
tr|Q43006|Q43006_ORYSA	gi|20286|emb	Peroxidase	4.58	4.923	1.16	1.421	1.233
tr|Q5Z7J7|Q5Z7J7_ORYSJ	gi|55701041	Peroxidase	5.025	5.222	0.802	2.469	2.659
tr|Q25AK7|Q25AK7_ORYSA	gi|90265065	H0510A06.15 protein	1.326	1.047	0.91	1.209	1.022
tr|Q6K4J4|Q6K4J4_ORYSJ	gi|115479691	Peroxidase	1.23	1.049	0.988	1.16	0.919
tr|A2WJQ7|A2WJQ7_ORYSI	gi|115434036	Os01g0106400 protein	0.884	2.12	0.973	1.313	2.057
sp|P41095|RLA0_ORYSJ	gi|115474653	60S acidic ribosomal protein	1.312	1.231	1.073	0.882	0.83
sp|B8AUI3|GLO3_ORYSI	gi|115460650	Peroxisomal (S)-2-hydroxy-acid oxidase GLO3	0.627	0.615	1.305	0.833	0.972
tr|A0A0N7KI36|A0A0N7KI36_ORYSJ	gi|55700967	Peroxidase	0.895	0.817	0.934	1.403	1.041
tr|B8B5W7|B8B5W7_ORYSI	gi|218200254	Peroxidase	1.11	1.51	0.996	2.708	1.966
tr|A2WPA1|A2WPA1_ORYSI	gi|125525683	Peroxidase	1.258	1.625	1.07	2.133	3.577
tr|A2ZAA6|A2ZAA6_ORYSI	gi|115483466	Putative peptide methionine sulfoxide reductase	1.121	1.184	0.902	1.739	1.32
tr|A2XVK6|A2XVK6_ORYSI	gi|125549044	Putative uncharacterized protein	0.844	0.93	0.946	1.321	1.19
tr|B9F688|B9F688_ORYSJ	gi|222624472	Uncharacterized protein	2.063	3.091	1.018	2.407	2.351
tr|B8AU10|B8AU10_ORYSI	gi|218194884	Putative uncharacterized protein	1.206	0.747	1.145	1.386	1.226
tr|Q7F1J9|Q7F1J9_ORYSJ	gi|115477368	Os08g0522400 protein	1.225	1.347	1.072	1.309	1.121
sp|Q6K471|FTRC_ORYSJ	gi|75125055	Ferredoxin-thioredoxin reductase	1.28	2.03	0.905	1.598	1.668
tr|A0A0B4U1V7|A0A0B4U1V7_ORYSA	gi|115467518	Aldehyde dehydrogenase ALDH2b	1.178	1.029	1.016	1.006	1.233
sp|Q6AV34|ARGC_ORYSJ	gi|218193315	Probable N-acetyl-gamma-glutamyl-phosphate reductase	1.046	1.075	0.971	0.966	1.237
tr|Q2QV45|Q2QV45_ORYSJ	gi|115487998	70 kDa heat shock protein	1.415	1.387	1.068	1.391	1.254
sp|Q84VG0|CML7_ORYSJ	gi|115474531	Putative uncharacterized protein	1.351	1.579	0.859	1.35	1.392
tr|A2Y8A8|A2Y8A8_ORYSI	gi|115465902	Os06g0104300 protein	0.877	2.193	1.078	1.336	1.654
tr|A0A0P0X7V0|A0A0P0X7V0_ORYSJ	gi|115472943	Os07g0573800 protein (Fragment)	1.61	1.898	0.916	1.161	1.207
tr|B8BAM3|B8BAM3_ORYSI	gi|115474739	Os08g0139200 protein	1.096	1.539	0.841	0.9	1.207
sp|Q69TY4|PR2E1_ORYSJ	gi|115469028	Putative uncharacterized protein	1.289	1.212	0.944	1.336	1.361
sp|Q8W3D9|PORB_ORYSJ	gi|75248671	Protochlorophyllide reductase B	0.881	1.621	0.891	1.192	2.065
tr|B8AGN1|B8AGN1_ORYSI	gi|115445869	Os02g0328300 protein	1.63	2.925	0.927	1.814	1.808
tr|B9F604|B9F604_ORYSJ	gi|222625905	Uncharacterized protein	1.474	1.82	1.086	1.62	1.613
tr|Q7F229|Q7F229_ORYSJ	gi|115471449	Os07g0260300 protein	0.924	1.084	0.949	1.375	2.104
tr|A6N0B2|A6N0B2_ORYSI	gi|149391329	Mitochondrial formate dehydrogenase 1 (Fragment)	0.993	1.084	0.931	0.99	1.212
sp|Q10L32|MSRB5_ORYSJ	gi|115453111	Putative uncharacterized protein	1.116	1.471	0.84	1.272	1.479
tr|Q941T6|Q941T6_ORYSJ	gi|15408884	Os01g0847700 protein	1.028	0.871	1.18	1.416	1.504
tr|B8B2F2|B8B2F2_ORYSI	gi|218198209	Formate dehydrogenase	1.014	1.19	0.97	0.898	1.278
sp|Q7XPL2|HEM6_ORYSJ	gi|75232919	OSIGBa0152L12.9 protein	0.993	1.224	0.873	0.963	1.253
sp|P0C5D4|PRXQ_ORYSI	gi|115466906	Peroxiredoxin Q, chloroplastic	1.215	1.691	0.909	1.71	2.077
tr|A0A0P0WR9|A0A0P0WWR9_ORYSJ	gi|300681235	Os06g0472000 protein	1.308	1.269	1.032	1.532	1.593
tr|A2WL79|A2WL79_ORYSI	gi|125524611	Peroxidase	0.826	0.852	1.047	1.182	1.233
sp|P37834|PER1_ORYSJ	gi|115464711	Peroxidase	0.702	0.999	0.819	0.742	1.764
tr|Q01LB1|Q01LB1_ORYSA	gi|115458104	OSJNBa0072K14.5 protein	1.175	1.243	0.937	1.124	1.221
sp|P0C0L1|APX6_ORYSJ	gi|115487636	Putative uncharacterized protein	1.127	1.213	1.031	1.282	1.477
sp|Q7X8R5|TRXM2_ORYSJ	gi|115459582	B1011H02.3 protein	1.557	2.198	0.916	1.577	3.486
tr|B7E4J4|B7E4J4_ORYSJ	gi|215704355	Putative uncharacterized protein	0.853	1.062	0.762	0.579	1.105
tr|Q7XV08|Q7XV08_ORYSJ	gi|38567882	OSJNBa0036B21.10 protein	1.159	1.382	0.971	1.084	1.397
**Carbohydrate metabolism**							
sp|Q8L7J2|BGL06_ORYSJ	gi|218192323	Beta-glucosidase 6	0.177	0.383	1.004	0.424	1.741
sp|Q76BW5|XTH8_ORYSJ	gi|115475445	Xyloglucan endotransglycosylase/hydrolase protein 8	0.953	2.101	0.939	1.074	1.369
tr|Q01JC3|Q01JC3_ORYSA	gi|116310134	Malate dehydrogenase	0.795	0.74	0.995	0.591	0.889
tr|Q0DCB1|Q0DCB1_ORYSJ	gi|115467998	Os06g0356700 protein	0.849	1.073	0.912	1.227	2.764
tr|Q10CU4|Q10CU4_ORYSJ	gi|115455353	GH family 3 N terminal domain containing protein, expressed	0.72	2.799	0.66	0.663	2.234
tr|Q9ZNZ1|Q9ZNZ1_ORYSA	gi|4097938	Beta-1,3-glucanase	0.795	1.711	1.003	0.932	1.619
tr|H2KWT0|H2KWT0_ORYSJ	gi|108863034	HIPL1 protein, putative, expressed	1.106	2.099	0.908	1.231	2.014
tr|B8AIS2|B8AIS2_ORYSI	gi|218191593	Putative uncharacterized protein	0.773	0.837	0.875	1.437	1.452
sp|Q0INM3|BGA15_ORYSJ	gi|115488372	Beta-galactosidase 15	1.348	1.602	0.924	1.187	1.56
tr|B9FWS5|B9FWS5_ORYSJ	gi|222636880	Uncharacterized protein	0.838	1.177	1.122	0.866	1.141
tr|Q0JG30|Q0JG30_ORYSJ	gi|297598314	Os01g0946500 protein	0.95	0.844	0.995	0.799	0.959
tr|Q0J0Q9|Q0J0Q9_ORYSJ	gi|115479865	Os09g0487600 protein	0.829	1.294	0.88	1.272	1.594
tr|A2XM08|A2XM08_ORYSI	gi|115455349	GH family 3 N terminal domain containing protein, expressed	0.859	1.124	0.866	0.78	1.387
sp|Q10NX8|BGAL6_ORYSJ	gi|152013362	Beta-galactosidase 6	1.063	1.684	0.938	1.413	1.814
tr|B8AII1|B8AII1_ORYSI	gi|218190145	Putative uncharacterized protein	0.904	1.55	0.954	1.167	1.478
tr|Q01IH0|Q01IH0_ORYSA	gi|116310092	H0502G05.3 protein	0.728	0.783	0.894	1.001	1.193
tr|Q01JK3|Q01JK3_ORYSA	gi|116310050	Aldose 1-epimerase	0.823	1.226	0.939	1.515	1.515
tr|B8BHM7|B8BHM7_ORYSI	gi|10140702	Alpha-galactosidase	0.723	1.32	1.006	1.331	1.544
tr|A2Z9V6|A2Z9V6_ORYSI	gi|125532825	Uncharacterized protein	0.73	2.017	0.876	1.276	1.194
tr|Q0DTS9|Q0DTS9_ORYSJ	gi|297600575	Os03g0227400 protein (Fragment)	1.101	1.226	0.804	1.06	1.306
tr|A2XME9|A2XME9_ORYSI	gi|115455637	Malate dehydrogenase	1.049	1.261	1.151	1.502	1.548
tr|Q6Z8F4|Q6Z8F4_ORYSJ	gi|115448091	Phosphoribulokinase	1.143	1.318	1.066	1.144	1.249
tr|A2YIJ5|A2YIJ5_ORYSI	gi|50509727	Os07g0168600 protein	0.779	0.93	0.952	1.118	1.317
sp|Q75I93|BGL07_ORYSJ	gi|115454825	Beta-glucosidase	1.201	1.066	0.95	1.276	1.58
tr|Q7XIV4|Q7XIV4_ORYSJ	gi|115474081	Alpha-galactosidase	0.786	1.367	0.919	1.115	1.491
tr|A3A285|A3A285_ORYSJ	gi|115443693	Uncharacterized protein	0.83	1.101	0.843	1.151	1.262
tr|A0A0P0XVT5|A0A0P0XVT5_ORYSJ	gi|297610712	Alpha-galactosidase (Fragment)	0.72	1.115	0.848	1.16	1.321
tr|B7F946|B7F946_ORYSJ	gi|297605789	Os06g0356800 protein	0.681	1.016	0.7	1.159	2.984
**Stress responsive**							
tr|Q9AQU0|Q9AQU0_ORYSJ	gi|13486733	Peptidyl-prolyl cis-trans isomerase	1.249	1.825	0.965	1.578	1.772
tr|Q8GTB0|Q8GTB0_ORYSJ	gi|27476086	Putative heat shock 70 KD protein, mitochondrial	1.294	1.354	0.92	1.027	1.208
tr|Q84S20|Q84S20_ORYSJ	gi|28971968	CHP-rich zinc finger protein-like	2.605	2.416	0.869	1.374	1.439
tr|Q5JKK9|Q5JKK9_ORYSJ	gi|115442153	Os01g0940700 protein	1.897	3.948	0.955	1.04	0.959
sp|Q75HQ0|BIP4_ORYSJ	gi|115464027	Heat shock 70 kDa protein BIP4	10	10	1.058	0.8	0.72
tr|Q53NM9|Q53NM9_ORYSJ	gi|115486793	DnaK-type molecular chaperone hsp70-rice	1.87	1.487	1.009	0.821	0.793
tr|Q10NA9|Q10NA9_ORYSJ	gi|115452223	70 kDa heat shock protein	2.198	1.668	1.086	0.907	0.816
sp|Q5VRY1|HSP18_ORYSJ	gi|115434946	17.5 kDa heat shock protein	1.413	3.508	1.045	1.084	1.043
tr|Q6YUA7|Q6YUA7_ORYSJ	gi|115476792	Os08g0464000 protein	1.323	1.3	1.041	0.866	1.034
tr|A2YK26|A2YK26_ORYSI	gi|115471453	Os07g0262200 protein	1.096	1.252	0.994	0.995	1.314
tr|B9FK56|B9FK56_ORYSJ	gi|222631026	Uncharacterized protein	1.028	1.106	1.031	1.314	1.25
tr|A2Z3L9|A2Z3L9_ORYSI	gi|115480445	Os09g0541700 protein	1.1	1.218	0.999	1.144	1.342
tr|O82143|O82143_ORYSJ	gi|115451853	26S proteasome regulatory particle	1.138	1.146	0.981	1.293	1.427
tr|Q5ZAV7|Q5ZAV7_ORYSJ	gi|115440349	Os01g0783500 protein	1.066	1.434	1.03	1.71	2.189
tr|A2Y628|A2Y628_ORYSI	gi|125552829	Cysteine proteinase inhibitor	0.96	1.775	1.056	1.438	2.205
**Osmotic stress responsive**							
tr|A2XHR1|A2XHR1_ORYSI	gi|125544232	Sucrose synthase	0.82	1.102	0.545	0.366	1.005
tr|B8B835|B8B835_ORYSI	gi|115473055	NADH-dehydrogenase	0.992	1.182	0.879	1.282	1.538
tr|Q2RBD1|Q2RBD1_ORYSJ	gi|115483847	Non-specific lipid-transfer protein	0.988	1.244	0.894	1.274	2.009
tr|Q0IQK7|Q0IQK7_ORYSJ	gi|297612544	Non-specific lipid-transfer protein	1.226	2.979	0.78	1.023	2.235
tr|B8B936|B8B936_ORYSI	gi|218201512	Putative uncharacterized protein	0.871	1.281	0.93	0.976	1.51
tr|B8AII1|B8AII1_ORYSI	gi|218190145	Putative uncharacterized protein	0.904	1.55	0.954	1.167	1.478
tr|Q9SNL7|Q9SNL7_ORYSJ	gi|6006382	Putative SAM-protoporphyrin IX methyltransferase	0.935	0.974	0.965	1.05	1.216
sp|Q10LR9|DCUP2_ORYSJ	gi|115452897	Uroporphyrinogen decarboxylase 2	1.265	1.835	0.902	0.95	1.425
tr|A2X8B7|A2X8B7_ORYSI	gi|242062934	2-C-methyl-d-erythritol 2,4-cyclodiphosphate synthase	1.362	1.399	0.746	1.202	1.538
tr|Q2RBD1|Q2RBD1_ORYSJ	gi|115483847	Non-specific lipid-transfer protein	0.988	1.244	0.894	1.274	2.009
**Ethylene responsive**							
tr|B9G3V3|B9G3V3_ORYSJ	gi|222641669	Uncharacterized protein	1.837	2.313	1.837	1.825	1.982
sp|Q8W3D9|PORB_ORYSJ	gi|75248671	Protochlorophyllide reductase B	0.881	1.621	0.891	1.192	2.065
tr|Q0IQK7|Q0IQK7_ORYSJ	gi|297612544	Non-specific lipid-transfer protein (Fragment)	1.226	2.979	0.78	1.023	2.235
tr|Q2RBD1|Q2RBD1_ORYSJ	gi|115483847	Non-specific lipid-transfer protein	0.988	1.244	0.894	1.274	2.009
**Metabolic responsive**							
tr|Q0D572|Q0D572_ORYSJ	gi|297607511	Os07g0577300 protein	1.28	1.719	1.105	0.899	2.422
tr|A2YIJ5|A2YIJ5_ORYSI	gi|50509727	Os07g0168600 protein	0.779	0.93	0.952	1.118	1.317
tr|B9F240|B9F240_ORYSJ	gi|222622048	Uncharacterized protein	0.739	1.149	1.372	1.24	1.422
tr|B9F7T1|B9F7T1_ORYSJ	gi|222624734	Uncharacterized protein	1.389	1.083	1.317	0.854	0.954

## Abbreviations

LYP9	Liangyoupeijiu
NPBA	Nipponbare
iTRAQ	Isobaric tags for relative and absolute quantitation
CMI	Cell membrane injury
RRA	Rice root activity
DEPs	Differentially expressed proteins
GO	Gene ontology
KEGG	Kyoto encyclopedia of genes and genomes
PSI	Photosystem I
LS	Low salt stress
MS	Moderate salt stress
HS	High salt stress
COG	Cluster of orthologous groups
